# Therapeutic empathy in remote consultations in general practice: a realist review protocol

**DOI:** 10.1136/bmjopen-2026-119075

**Published:** 2026-05-14

**Authors:** Jeremy Howick, Karen Ma, Amber Bennett-Weston, Andy Ward, Nia Roberts, Jennifer Bostock, Jono Broad, Geoff Wong

**Affiliations:** 1 Stoneygate Centre for Empathic Healthcare, Leicester Medical School, University of Leicester, Leicester, UK; 2 Bodleian Health Care Libraries, University of Oxford, Oxford, UK; 3 Care Policy Evaluation Center, The London School of Economics and Political Science, London, UK; 4 South West Primary Community Personalised Care, NHS England, Taunton, UK; 5 Nuffield Department of Primary Care Health Sciences, University of Oxford, Oxford, UK

**Keywords:** General Practice, Empathy, Patient-Centered Care, Telemedicine

## Abstract

**Introduction:**

Remote consultations (video, telephone, text) have become integral to the delivery of primary care and are promoted by government initiatives. While many find these more convenient, they may also discriminate against those with lower digital literacy and present a barrier to empathy by removing some non-verbal communication. The aim of this realist review is to understand how therapeutic empathy can be effectively expressed during remote consultations in general practice across different situations and for different people.

**Methods and analysis:**

This realist review will follow the methodological framework proposed by Pawson and colleagues, which includes the following five steps: (1) identify existing theories to develop an initial programme theory; (2) systematically search bibliographic databases to identify relevant literature; (3) select, extract and organise data; (4) synthesise evidence to develop context-mechanism-outcome configurations; (5) refine and finalise programme theory. This iterative process will be guided by a Content Expert Group consisting of patients, carers, clinical staff working in general practice and representatives from national stakeholder groups. The final programme theory will inform the development of evidence-based recommendations to help clinical staff working in general practice express empathy during remote consultations.

**Ethics and dissemination:**

This review does not require ethics approval. Findings will be disseminated through peer-reviewed journals, national and international conferences and through relevant professional associations and primary care networks in the UK.

**PROSPERO registration number:**

CRD420261306014.

**STRENGTHS AND LIMITATIONS OF THIS STUDY**

- Realist methodology addresses a gap in the literature, namely understanding how therapeutic empathy might be delivered in remote consultations with diverse patient populations.
- Ongoing involvement of patients, carers, clinical staff working in general practice and national stakeholders throughout the review will support the relevance, credibility and feasibility of the programme theory and recommendations.
- Including evidence from a wide range of sources (including qualitative studies, policy documents and grey literature) will enhance conceptual richness but may introduce variability in methodological quality and reporting.

## Introduction

 Therapeutic empathy involves healthcare practitioners understanding and sharing patients’ perspectives, responding emotionally and taking action, while maintaining professional boundaries.^1^ Randomised trials show that empathy can improve patient outcomes, such as pain reduction, anxiety management, satisfaction and enablement.^2^,^5^ Apart from improving patient care, therapeutic empathy can also improve practitioner well-being, as practitioners with high levels of empathy are less likely to report being burned out.^6^ While empathy is a learnable skill,^7^ it is inconsistently delivered in face-to-face consultations.^8^ A systematic review and meta-analysis of patient surveys found that female practitioners were more empathic than male practitioners and allied health professionals were the most empathic healthcare providers, whereas doctors were the least empathic.^9^ Such disparities in empathic care can result in serious harm, as numerous government reports found that lack of empathy caused avoidable adverse events.^10 11^

Whereas empathy in healthcare is widely studied in in-person consultations, an increasing number of primary care consultations are delivered remotely (eg, via video, telephone and texts). In the UK, the National Health Service (NHS) Long Term Plan has mandated digital-first primary care since 2023/24.^12^ Following the policy proposal, the proportion of remote consultations delivered through video/online has increased from 0.5% in 2022 to 5.7% in 2024, which translates to approximately 9 million primary care appointments occurring remotely via online consultation tools or telephone/video consultations in December 2024 alone.^13^ While remote consultations may improve access to care through increased convenience and flexibility, they may introduce barriers including calls at inconvenient times, technical difficulties and digital exclusion.^14 15^ This may create inequities for patients with limited digital literacy, lack of access to appropriate technology or unreliable internet connectivity, as well as for those from cultures that may be less comfortable with remote consultations.

In terms of patient-practitioner interactions, remote consultations also potentially pose additional challenges to delivering therapeutic empathy by removing non-verbal communication channels such as eye contact, facial expressions and posture.^16 17^ Recently, a rapid systematic review found that expressing therapeutic empathy in telephone consultations is possible; however, general practitioners expressed low confidence and concerns about how to express empathy in this format, with insufficient research to support them.^17^ Research has also found that patients consistently rated therapeutic empathy lower in telephone consultations when compared with face-to-face consultations.^15^ Moreover, nurses working in general practice also raised concerns about remote consultations diminishing relational and therapeutic dimensions of care, with care becoming more transactional and fragmented.^18^ Physiotherapists providing musculoskeletal care in general practice also expressed difficulty building rapport with patients in the lack of physical presence and how this hinders their abilities to express empathy.^19^ Therefore, it is unclear how clinical staff working in general practice can best express therapeutic empathy during remote consultations.

The issue of therapeutic empathy in remote consultations is further complicated by the fact that the expression and reception of empathy depends on patient and practitioner characteristics. A recent systematic review found that empathy is particularly lacking for marginalised populations, including those with lower socioeconomic status or from ethnic minorities.^20^ Another quantitative study also revealed that people with disabilities experienced negative implicit bias from practitioners (n=25 006).^21^ As general practice delivers primary care services to a highly heterogeneous patient population, care must be responsive to diverse age, socioeconomic status, culture and ethnicity, as well as digital literacy. As the expression of empathy is influenced by multiple interacting contextual factors, the realist review methodology provides a theory-driven approach to understand and explain how, for whom and in what contexts therapeutic empathy can be expressed during remote consultations in general practice. As the realist methodology enables the examination of context-mechanism-outcome relationships and allows us to develop causal, mechanistic understanding, it is well suited to address the complexity and heterogeneity of remote consultations and patient populations.

### Aims and objectives

The aim of this realist review is to develop a programme theory to explain how clinical staff working in general practice can express therapeutic empathy in remote consultations across different contexts and for different people. The resulting programme theory will be used to develop evidence-based recommendations for clinical staff working in general practice that are actionable, inclusive and context-sensitive, and will benefit all NHS primary care patients in the UK.

## Methods and analysis

**Figure 1:**
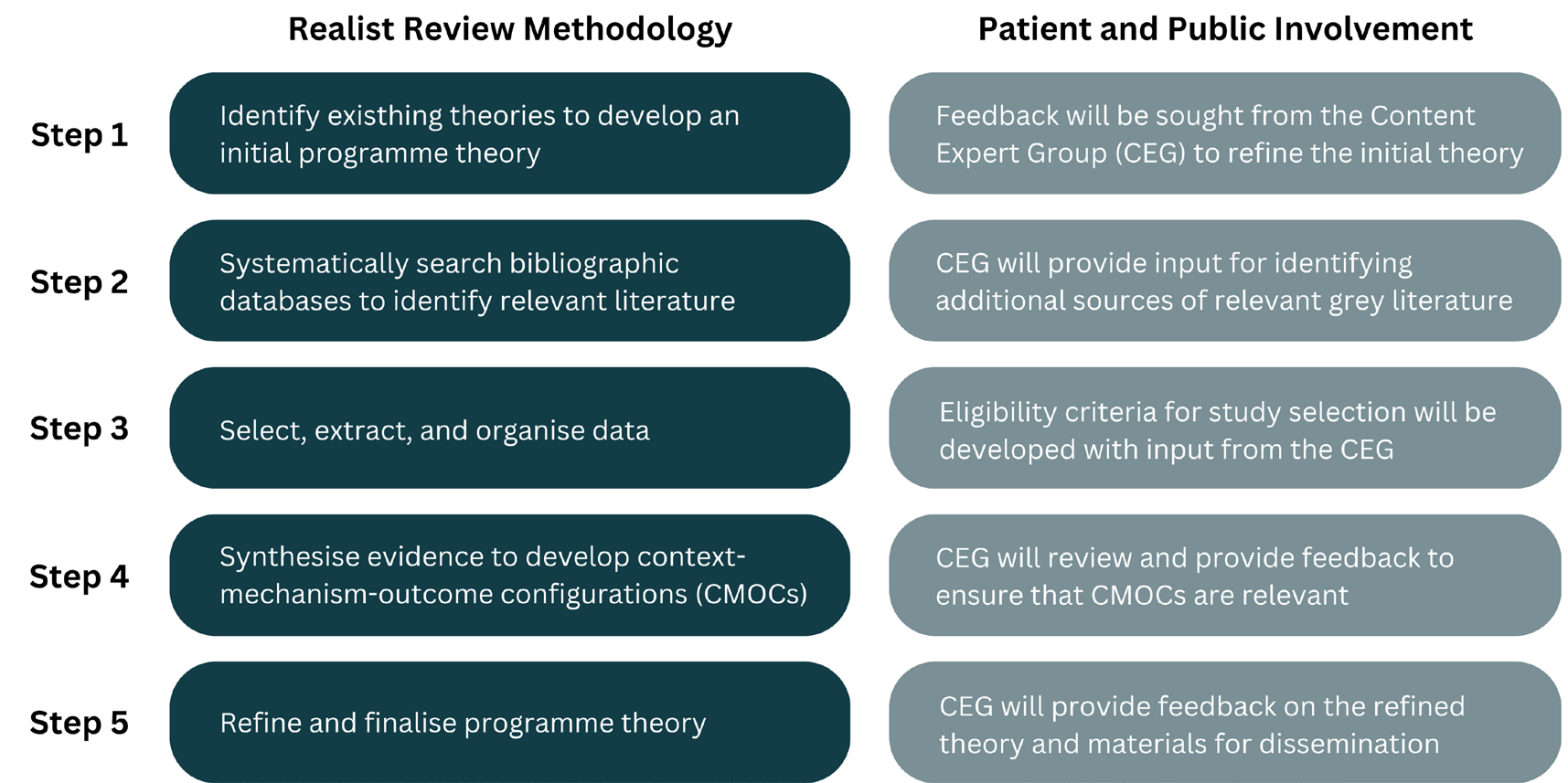
Outline of the realist review methodology with input from patient and public involvement. CEG, Content Expert Group; CMOCs, context-mechanism-outcome-configurations.

This realist review will be guided by the methodological framework proposed by Pawson and colleagues, which consists of an iterative, five-step method to understand what works for whom and in what circumstances^22^ (figure 1). We will systematically gather existing evidence and build a realist programme theory that contains causal explanations in the form of context-mechanism-outcome-configurations (CMOCs). The review will also follow the Realist and Meta-narrative Evidence Syntheses: Evolving Standards (RAMESES) quality and reporting standards for realist synthesis.^23^ This protocol was prospectively registered with PROSPERO in February 2026 (Registration number CRD420261306014). The review commenced in March 2026 and is anticipated to be completed by 2027.

### Patient and public involvement

This protocol was developed with input from a diverse, UK-wide Patient Advisory Group (PAG) recruited via local patient and carer groups and the National Institute of Health and Care Research (NIHR) People in Research website.^24^ The PAG comprised eight individuals from different ethnic, age and socioeconomic backgrounds, with lived experience of various physical and mental health conditions, neurodiversity, disability, as well as experiences of remote consultations, as patients, carers and/or parents. Based on the recommendations of the PAG, we decided to focus on primary care settings, as remote consultations are more common in primary care and affect more patients, thereby increasing the impact of this work. Moreover, as primary care is where the patient journey starts, PAG members emphasised that whether a remote primary care consultation is empathic could impact experiences and outcomes in secondary care. This protocol is co-authored by two of the PAG members (JBo and JBr).

In order to incorporate lived experiences and ensure the review’s relevance in real-world settings, the PAG will continue to provide feedback on the review findings and support output production and dissemination. Before commencing the review, we will expand the PAG to include up to 12 members, including two teenagers to incorporate their voice in our research. To ensure that teenagers’ voices are heard and avoid creating a potential power imbalance with adult PAG members, we will enable them to decide how they wish to be involved, whether they are consulted by the research team separate from PAG meetings and exchanges, or included in the PAG meetings and exchanges, or a combination of these. We will also adopt a flexible engagement approach, such as one-to-one calls where preferred by a PAG member, joint attendance with a carer or family member and the ability to receive or send information verbally where preferred due to low literacy or low English-language levels to support wide inclusion. The PAG will meet the research team regularly and will also join professional stakeholders in the Content Expert Group (CEG) to guide the review (see next section). Public contributors will be reimbursed following the NIHR Reward and Recognition guidance.^25^

### Joint contributions with professional stakeholders

Throughout the review, the research team will work with patient, public and professional stakeholders in a nationwide CEG. The CEG will comprise PAG members, clinical staff working in general practice, empathy experts and representatives from national stakeholder groups, including NHS England, Integrated Care Boards (ICBs), Royal College of General Practitioners (RCGP) and Care Quality Commission (CQC). An equality impact assessment will be undertaken to support the recruitment of a diverse CEG through including contributors from excluded populations and intersectionally excluded populations. We will first introduce the project and explain key concepts of the realist review methodology in clear, accessible terms using visuals and creative analogies. Stakeholders will then provide feedback on the development of the initial programme theory, refinement of the eligibility criteria, review of the refined programme theory and planning of dissemination to ensure that the programme theory is fit-for-purpose and can be realistically implemented within the NHS (see figure 1). The CEG will meet online regularly (initially planned to be monthly) throughout the course of the review, with continued communication between meetings to maintain cohesion. Flexible engagement approaches will also be adopted to support wider inclusion. In case of CEG members leaving during the review, replacement members will be recruited to maintain diversity of views.

### Step 1: Develop initial programme theory

The research team will first develop an initial programme theory based on a preliminary exploratory search of the literature to identify any existing theories relevant to delivering therapeutic empathy in remote consultations. We anticipate including theories of empathy and therapeutic communication, computer-mediated communication and digital health implementation, as well as the literature on empathy in face-to-face consultations, remote consultation delivery and barriers and facilitators to empathy. The initial programme theory will be developed through research team meetings, leveraging team expertise in empathy and primary care including their knowledge and contacts to identify relevant additional sources of information. The initial theory will be presented to the CEG for feedback and refinement. Together, the research team and the CEG will generate a visual map and narrative of initial theories showing (where possible) hypothesised relationships between contexts, mechanisms and outcomes to be tested throughout the review process.

### Step 2: Conduct evidence search

#### Search strategy

A formal search of the literature will be conducted through electronic databases and grey literature sources. Eight electronic databases will be searched, including MEDLINE, EMBASE, PsycINFO, CINAHL, Scopus, Web of Science, Cochrane Library and ProQuest Dissertations and Theses. Relevant grey literature will be identified through Overton, alongside a structured search of websites of relevant organisations (eg, RCGP, NHS England, ICBs, CQC, patient organisations) for policy documents and clinical guidelines. Forward and backward citation searching of included documents will be undertaken to identify additional relevant documents. Search strategies will be developed and piloted by the lead reviewer (KM) with the support of an information specialist (NR) and will include both free text and controlled vocabulary terms. We expect to use search terms such as ‘empathy’, ‘patient-centred communication’, ‘remote’, ‘digital’, ‘online’, ‘video’, ‘telephone’, ‘messaging’, ‘general practice’, ‘primary care’, ‘consultation’ and ‘appointment’. Additional searches will be undertaken throughout the review as new information requirements emerge, continuing until theoretical saturation occurs and the refined programme theory is coherent and plausible. An example of the full search strategy is provided in the online supplemental materials.

#### Eligibility criteria

The inclusion and exclusion criteria will be developed with input from the CEG. Initially, we anticipate including documents that explored empathy as the main outcome, conducted in general practice where remote consultations were utilised and involved any combination of patients, carers and parents of patients. Consultations with CEG will begin with a clarification of what ‘therapeutic empathy’ and ‘remote consultations’ encompass, where ‘remote consultations’ are defined as ‘healthcare consultations’ delivered through ‘remote formats’. We suggest the following criteria as a starting point for discussion with CEG:

##### Therapeutic empathy (based on Howick *et al*^1^):

- Exploring patient’s experiences, emotions and expectations.
- Understanding patient’s perspectives and circumstances through cognitive processes.
- Shared understanding of patient’s circumstance, values, preferences and options.
- Feelings experienced by practitioners in response to understanding patients’ perspectives.
- Therapeutic actions, which are caring behaviours to help patients.
- Maintaining boundaries throughout the patient-practitioner interaction.

##### Healthcare consultations:

- Synchronous, two-way interactions between patients and practitioners.
- Only where both patients and practitioners are humans.
- Regarding patients’ physical, mental and social health (including assessment and management, excluding administrative matters).

##### Remote formats of delivery:

- Video (audio-visual).
- Telephone (audio only).
- Live chat or secure text messaging (text-based).

With regard to the study designs, peer-reviewed primary studies (including quantitative, qualitative and mixed-methods studies) and dissertations will be included, as well as any policy documents and clinical guidelines identified in the grey literature. While systematic reviews and conference abstracts will be excluded, reference lists of related reviews and abstracts will be searched for potentially relevant documents.

### Step 3: Select, extract and organise data

#### Study selection

Search results will be managed in Covidence. All records will be screened against the predefined eligibility criteria in two stages (title and abstract screening, followed by full text screening). At both stages, all records will be screened by the lead reviewer (KM), and a randomly selected subsample of a minimum of 10% of the records will be screened independently by a second reviewer to detect systematic errors; and any disagreements will be resolved by discussion.

#### Quality appraisal

All documents will be appraised by the lead reviewer (KM), and a minimum of 10% random subsample will be appraised independently by a second reviewer; and any disagreements will be resolved by discussion. Documents will not be excluded based on methodological quality alone. Instead, we will appraise documents for relevance and, where necessary, rigour.^26^ Relevance will be assessed based on whether documents contain data that contribute to programme theory development and refinement, whereas rigour will be assessed based on whether methods used to generate the data are credible (eg, appropriate for the research question, transparent, systematic), and whether the inferences drawn from the data are trustworthy (eg, sufficiently explained, supported by data, acknowledge limitations). Assessments of rigour will only take place in certain situations, for example when a document contributes what we judge to be a substantial amount of relevant data. Rigour will be assessed by a series of guiding questions regarding the relevant data (rather than the study as a whole), including whether the included data are likely to be biased, whether they were dealt with critically, whether they were from a real-world sample or theoretical speculation, whether the data were gathered in some depth over time, and whether it is safe to generalise from these data.^27^ We will judge the plausibility and coherence of the explanation provided by the programme theory using three criteria: consilience (whether multiple independent sources support the same CMOC), simplicity (whether explanations are parsimonious) and analogy (whether our findings ‘fit in’ with existing knowledge).^26^ We will also classify the documents using the process set out by Dada and colleagues^28^ into whether a document provides major or minor contributions. This approach will help us focus initially on extracting and analysing data from documents that provide important, conceptually rich contribution, while still retaining useful insights from the wider literature. All included documents will eventually be analysed—starting first from those making a major contribution then later on to minor ones. Included documents will be classed as providing major or minor contributions to the research questions according to the following provisional criteria:

##### Major contributions

- Documents that contribute relevant data to the research questions and are conducted in the NHS or in healthcare systems with similarities to the NHS.
- Documents that contribute relevant data to the research questions and are judged to clearly help to identify mechanisms that could plausibly operate in the circumstances of the NHS.

##### Minor contributions

- Documents conducted in healthcare systems that are markedly different to the NHS but where the mechanisms could plausibly operate in the NHS.
- Documents where clinical staff working in general practice form a small proportion of the population studied.

### Data extraction

All data will be extracted by the lead reviewer (KM), and a 10% random subsample will be extracted independently by a second reviewer; and any disagreements will be resolved by discussion. For the descriptive characteristics of included documents, a data extraction form will be developed and piloted on at least five randomly selected articles. This will be used to extract the following characteristics of the included documents: authors; publication year; country; study design and methods; setting; consultation modality (eg, video, telephone, text); population characteristics (including demographics relevant to excluded populations). For the data relevant to programme theory development, full texts will be uploaded into NVivo for coding and organisation of data. Coding will involve extracting relevant sections of the text from included documents that contribute to the building and testing of programme theory, which will be deductive (informed by initial programme theory), inductive (coming from data within included documents) and retroductive (making inferences based on interpretations of the data about underlying causal processes and mechanisms).

### Step 4: Synthesise evidence to develop context-mechanism-outcome configurations

Extracted data will be synthesised using a realist logic of analysis to build realist causal explanations, expressed as CMOCs. These will explain why clinical staff working in general practice find it hard to express empathy during remote consultations and identify any suggestions or techniques that clinical staff working in general practice have found useful in helping them express therapeutic empathy in remote consultations across different situations and populations. Specifically, we will consider whether data can be used to develop understanding that can be transferred to excluded and intersectionally excluded populations, or whether separate theories and/or additional data are needed to ensure relevance for diverse populations. This will include explicit consideration of socioeconomically deprived populations, ethnic minorities, LGBTQ+populations, older adults, people with disabilities, people with visual/hearing loss, and those with low English language proficiency or experiencing digital exclusion.

During the synthesis, we will make interpretations and judgements about the relationships between contexts, mechanisms, and outcomes by drawing on data from both within and across the included documents. Within each individual document, we will look for data that we can draw on to understand how contexts trigger mechanisms to produce outcomes. We will look to refine CMOCs by considering further data from other included documents (eg, mechanisms inferred from one document may help explain how contexts influenced outcomes in another). This is important and often necessary for building CMOCs as not all parts of the configurations will always be inferred from the same document. When making sense of our data, we will apply the following analytic strategies in line with the realist review methodology^29^:

- Juxtaposition of data sources: where data from one document provide insights into data from other documents.
- Reconciliation of conflicting data: where data differ in apparently similar contexts, further investigation can be conducted to explain why these differences have occurred.
- Adjudication of data: based on the methodological strengths or weaknesses of individual documents.
- Consolidation of data: where outcomes differ in particular contexts, an explanation can be constructed of how and why these outcomes occur differently.

### Step 5: Refine programme theory

Throughout the synthesis, the programme theory will be refined iteratively through testing (whether evidence support, refute or refine existing theoretical propositions), expansion (identifying new contexts, mechanisms and outcomes emerging from the evidence), consolidation (considering whether multiple CMOCs can be integrated into higher-order theoretical propositions) and specification (of the specific conditions under which the CMOCs operate). To support the programme theory refinement, we will assess the plausibility and coherence of our programme theory based on its consilience, simplicity and analogy (see Step 3 for more details).^26^

Furthermore, to ensure that the programme theory reflects clinical staff working in general practice, patients and other stakeholders’ experiences, the refined programme theory will be presented to the CEG for feedback. Specifically, we will try to understand whether the theory aligns with their experiences and identify any important contexts, mechanisms or outcomes that may be missing, or any aspects of the theory that seem implausible or need refinement. We will address any remaining gaps through targeted literature searches, stakeholder consultation and theoretical elaboration. This process will be repeated until no new information is provided by the evidence or patient and public involvement (PPI), or until theoretical saturation occurs and the final programme theory is coherent and plausible. Alongside key CMOCs summarised in a table, a visual representation of the final programme theory will be created to illustrate causal pathways between contexts, mechanisms and outcomes.

## Ethics and dissemination

This literature review will be a realist synthesis of the existing literature and will not collect any primary data and therefore does not require ethics approval. Although formal ethical approval is not required, we recognise that discussing healthcare consultations and empathy may involve personal stories during our PPI work; hence, we shall be steered by our PPI lead who is also a chair of a research ethics committee to ensure that such conversations are handled sensitively. Findings of the realist review will be disseminated through publications in peer-reviewed journals and presentations at national and international conferences. Apart from conventional academic dissemination, we will also disseminate our findings to clinical staff working in general practice in the UK through relevant professional associations and primary care networks. This will be done by translating the final programme theory into evidence-based recommendations systematically as guided by the Knowledge-to-Action framework^30^ and the GRADE-ADOLOPMENT approach.^31^ A practical implementation guide will be coproduced with the CEG, presented in the form of plain English summaries for public dissemination, infographics for rapid dissemination and other innovative formats for social media dissemination. This will address the current lack of guidelines or training to help clinical staff working in general practice express empathy during remote consultations, which is essential in light of NHS England’s prioritisation of digital-first healthcare and the impact of empathy on patient and practitioner outcomes.

## Supplementary material

10.1136/bmjopen-2026-119075 online supplemental file 1
