# Supplementary material for: Therapeutic empathy in remote consultations in general practice: a realist review protocol
Source: BMJ Open. 2026 May 14;16(5):e119075. doi: 10.1136/bmjopen-2026-119075 (PMC13182367; doi:10.1136/bmjopen-2026-119075)
Supplement: online supplemental file 1 [file bmjopen-16-5-s001.docx]

**Supplementary Material**

Example of draft search strategy for MEDLINE.

1 exp General Practice/

2 general practitioners/ or physicians, family/ or physicians, primary care/

3 Primary Health Care/

4 (gp or gps or ((general or family) adj3 (practi* or physician? or doctor?))).ti,ab,kf,jw. or bjgp.jw.

5 family medicine.ti,ab,kf,jw.

6 (primary adj3 (care or healthcare)).ti,ab,kf,jw.

7 ("out of hours" or ooh or nhs11 or nhs 111).ti,ab,kf.

8 (community health services/ or community mental health services/) and (allied health personnel/ or paramedics/ or nutritionists/ or occupational therapists/ or physical therapists/ or Physician Assistants/)

9 exp community health nursing/ or home nursing/ or home health nursing/

10 community pharmacy services/

11 (((family or community or home) adj5 (nurse? or therapist? or allied health professional? or physiotherapist? or physical therapist? or occupational therapist? or paramedic? or pharmacist? or dieti?ian? or mental health practitioner? or prescriber? or physician associate? or physician assistant?)) or district nurse? or health visitor?).ti,ab,kf.

12 1 or 2 or 3 or 4 or 5 or 6 or 7 or 8 or 9 or 10 or 11

13 Referral and Consultation/ or "Appointments and Schedules"/

14 (consult? or consultation? or communicat* or visit? or encounter? or appointment?).ti,kf.

15 13 or 14

16 Telemedicine/ or telephone/ or exp cell phone/ or videoconferencing/ or exp Internet/ or Mobile Applications/ or Telecommunications/ or Digital Health/

17 (tele* or phone? or cellphone? or smartphone? or video* or digital* or virtual* or online or electronic or web or internet or mobile or mhealth or m-health or ehealth or e-health).ti,kf.

18 16 or 17

19 15 and 18

20 Remote Consultation/

21 ((tele* or phone? or cellphone? or smartphone? or video* or digital* or virtual* or online or electronic or web or internet or mobile or mhealth or m-health or ehealth or e-health) adj5 (consult? or consultation? or communicat* or encounter? or visit? or appointment?)).ab.

22 (remote adj5 (consult? or consultation?)).ti,ab,kf.

23 (teleconsult* or teleconferen* or videoconsult* or videoconferen* or econsult* or e-consult* or evisit? or e-visit?).ti,ab,kf.

24 (telepharmac* or tele-pharmac*).ti,ab,kf.

25 (live chat or livechat or web chat or webchat or online chat or realtime chat or real-time chat or chat based care or chat based service? or facetime or instant messag* or whatsapp or text messag* or textmessag* or short messag* or sms).ti,ab,kf.

26 (((zoom or accurx or systmone or hero health or patchs nhs or patchs gp or patches nhs or patches nhs or patient access or babylon health or mygp or attendanywhere or drdoctor or wellola or t-pro or microsoft teams) adj5 (consult? or consultation? or visit? or appointment?)) or (engage consult or aire consult)).ti,ab,kf.

27 (telephone first or video first or digital first).ti,ab,kf.

28 19 or 20 or 21 or 22 or 23 or 24 or 25 or 26 or 27

29 Empathy/

30 Therapeutic Alliance/

31 Trust/

32 Communication Barriers/

33 empath*.ti,ab,kf.

34 (feel* adj5 (connected or acknowledged or understood or heard or listened)).ti,ab,kf.

35 (therapeutic adj (alliance? or relationship? or partnership? or interaction?)).ti,ab,kf.

36 (compassion* or trust or rapport).ti,ab,kf.

37 (communication adj3 (barrier? or obstacle? or challeng*)).ti,ab,kf.

38 29 or 30 or 31 or 32 or 33 or 34 or 35 or 36 or 37

39 12 and 28 and 38
