# Supplementary material for: Therapeutic empathy in remote consultations in general practice: a realist review protocol
Source: BMJ Open. 2026 May 14;16(5):e119075. doi: 10.1136/bmjopen-2026-119075 (PMC13182367; doi:10.1136/bmjopen-2026-119075)
Supplement: Supplementary file 2 [file bmjopen-2026-119075.reviewer_comments.pdf]

PEER REVIEW HISTORY

BMJ Open publishes all reviews undertaken for accepted manuscripts. Reviewers are asked to complete a checklist review form and are provided with free text boxes to elaborate on their assessment. These free text comments are reproduced below.

ARTICLE DETAILS

Title (Provisional)

Therapeutic empathy in remote consultations in general practice: A realist review protocol

Authors

Howick, Jeremy; Ma, Karen; Bennett-Weston, Amber; Ward, Andy; Roberts, Nia; Bostock, Jennifer; Broad, Jono; Wong, Geoff

---

VERSION 1 - REVIEW

**Reviewer**	**1**
**Name**	**Evans, Catrin**
**Affiliation**	**University of Nottingham, School of Health Sciences,**
**Date**	**09-Apr-2026**
**COI**	**None**

This is an excellent protocol on a highly important and relevant topic, with an excellent team and appropriate PPI and wider stakeholder input I have very few comments.

A few potential suggestions for further consideration are given below: -

I note that part of Step 2 (eligibility criteria) will involve defining "remote consultation" more precisely. This is important. I would urge the team to perhaps split this term even further to consider and create an operational definition for what is meant by "remote" and also what is meant by "consultation". The latter is particularly salient as, for example, some forms of online triage or instrumental online requests (e.g. for medications) may be variously understood as consultations - and different patient or professionals groups may have varying understandings of what "consultation" actually refers to.

I commend the team's approach to PPI and CEG involvement, and the support that is proposed to ensure meaningful inclusion. I wonder if more could be said about the training that may be required to help these groups to fully understand a realist approach. Some creative ways have been developed to communicate realist concepts to lay audiences, and I would be interested to see how the team is planning to approach this.

Finally, I felt that Step 1 was rather minimally explained. I think the protocol would benefit from more detail on the different elements of this Step.

VERSION 1 - AUTHOR RESPONSE

Comments	Authors' response (page numbers correspond to document with tracked changes)
**Reviewer 1 – Dr Catrin Evans**	
This is an excellent protocol on a highly important and relevant topic, with an excellent team and appropriate PPI and wider stakeholder input I have very few comments. A few potential suggestions for further consideration are given below.	Thank you for the positive comments regarding the overall quality of the protocol. We are grateful for your suggestions that are helpful for improving the review.
I note that part of Step 2 (eligibility criteria) will involve defining "remote consultation" more precisely. This is important. I would urge the team to perhaps split this term even further to consider and create an operational definition for what is meant by "remote" and also what is meant by "consultation". The latter is particularly salient as, for example, some forms of online triage or instrumental online requests (e.g. for medications) may be variously understood as consultations - and different patient or professionals groups may have varying understandings of what "consultation" actually refers to.	Page 10-11 lines 235-254<br>We have now separated the definition for "remote consultations" and specified what "healthcare consultations" and "remote formats of delivery" refer to respectively.
I commend the team's approach to PPI and CEG involvement, and the support that is proposed to ensure meaningful inclusion. I wonder if more could be said about the training that may be required to help these groups to fully understand a realist approach. Some creative ways have been developed to communicate realist concepts to lay audiences, and I would be interested to see how the team is planning to approach this.	Page 8 lines 189-191<br>We have specified that we will use visuals and creative analogies to explain realist review methodology to lay audiences.
Finally, I felt that Step 1 was rather minimally explained. I think the protocol would benefit from more detail on the different elements of this Step.	Page 9 lines 203-206<br>We have provided more details about the theories and literature we anticipate to include in the initial programme theory.
